# P-94. Can Fluoroquinolones Be Used for the Treatment of Deep-Seated *Streptococcus anginosus* group Infections?

**DOI:** 10.1093/ofid/ofae631.301

**Published:** 2025-01-29

**Authors:** Whitney Miller, Timothy C Jenkins, Joshua Parry, Sebastian Studnicka, Nicole Delino, Heather Young

**Affiliations:** Denver Health, Denver, Colorado; Denver Health, Denver, Colorado; Denver Health Medical Center, Denver, Colorado; Denver Health, Denver, Colorado; Denver Health, Denver, Colorado; Denver Health, Denver, Colorado

## Abstract

**Background:**

Invasive infections caused by *Streptococcus anginosus* group are increasingly common. Some experts recommend against treatment with fluoroquinolone (FQs) dues to potential development of fluoroquinolone resistance; however, this is not based on clinical data. The aim of this study is to compare the safety and effectiveness of FQ-based antibiotic regimens with non-FQ regimens for the treatment of deep-seated *S. anginosus* group infections.

**Figure:**
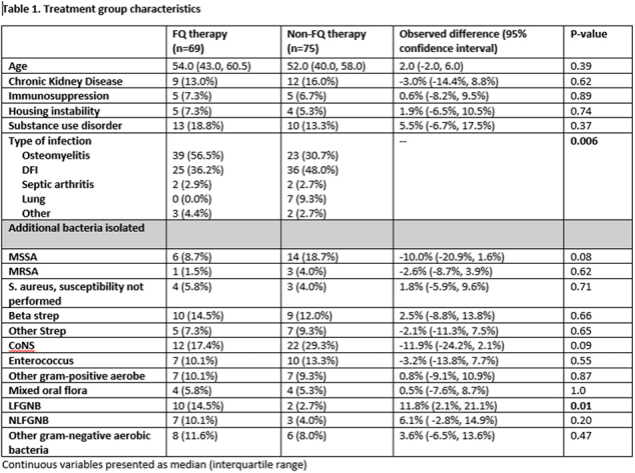

**Methods:**

This was a retrospective cohort study of patients hospitalized at Denver Health Medical center between 4/1/2016 and 10/31/2023 with a deep-seated infection and isolation of *S. anginosus* group from tissue, bone, synovial or pleural fluid. Cases were categorized as having been treated with a fluoroquinolone as part of their antibiotic regimen or not. The primary outcome was treatment success at 90 days as defined as absence of the following: recurrent infection, need for repeat surgical intervention or hospital readmission related to the primary infection. Secondary outcomes included antibiotic-related adverse events and central venous catheter complications.

**Figure:**
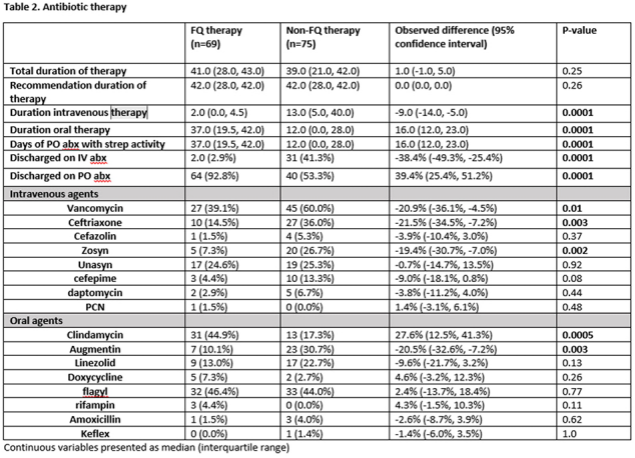

**Results:**

144 cases of deep-seated infection involving a *S. anginosus* group were identified; 69 received a FQ while 75 were treated with other antibiotics. Those in the FQ group were more likely to discharge on oral antibiotics (64 [93%] vs. 40 [53%], p< .001) and had a shorter duration of intravenous therapy (2 days vs. 13 days, p< .001). Treatment success occurred in 62 (90%) patients in the FQ group and 69 (92%) patients in the non-FQ group (p=0.65). Rates of antibiotic-related adverse events and vascular complications were similar between groups. Of patients who received a FQ, no patients had treatment failure or recurrent infection with an isolate resistant to a FQ.

**Figure:**
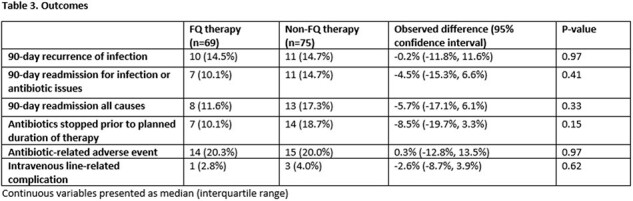

**Conclusion:**

FQs appeared to be a safe and effective treatment option for deep-seated *S. anginosus* group infections and facilitated early transition to oral therapy, while the development of FQ resistance was not observed.

**Disclosures:**

**All Authors**: No reported disclosures

